# The Distribution and Turnover of Bacterial Communities in the Root Zone of Seven *Stipa* Species Across an Arid and Semi-arid Steppe

**DOI:** 10.3389/fmicb.2021.782621

**Published:** 2021-12-24

**Authors:** Xiaodan Ma, Lumeng Chao, Jingpeng Li, Zhiying Ding, Siyu Wang, Fansheng Li, Yuying Bao

**Affiliations:** ^1^Key Laboratory of Forage and Endemic Crop Biotechnology, Ministry of Education, School of Life Sciences, Inner Mongolia University, Hohhot, China; ^2^State Key Laboratory of Reproductive Regulatory and Breeding of Grassland Livestock, Inner Mongolia University, Hohhot, China

**Keywords:** *Stipa* taxa, root-zone, bacterial communities turnover, assembly processes, environmental factor

## Abstract

The bacterial communities of the root-zone soil are capable of regulating vital biogeochemical cycles and the succession of plant growth. *Stipa* as grassland constructive species is restricted by the difference features of east–west humidity and north–south heat, which shows the population substituting distribution. The distribution, turnover, and potential driving factors and ecological significance of the root-zone bacterial community along broad spatial gradients of *Stipa* taxa transition remain unclear. This paper investigated seven *Stipa* species root-zone soils based on high-throughput sequencing combined with the measurements of multiple environmental parameters in arid and semi-arid steppe. The communities of soil bacteria in root zone had considerable turnover, and some regular variations in structure along the *Stipa* taxa transition are largely determined by climatic factors, vegetation coverage, and pH at a regional scale. Bacterial communities had a clear *Stipa* population specificity, but they were more strongly affected by the main annual precipitation, which resulted in a biogeographical distribution pattern along precipitation gradient, among which *Actinobacteria, Acidobacteria, Proteobacteria*, and *Chloroflexi* were the phyla that were most abundant. During the transformation of *Stipa* taxa from east to west, the trend of diversity shown by bacterial community in the root zone decreased first, and then increased sharply at *S. breviflora*, which was followed by continuous decreasing toward northwest afterwards. However, the richness and evenness showed an opposite trend, and α diversity had close association with altitude and pH. There would be specific and different bacterial taxa interactions in different *Stipa* species, in which *S. krylovii* had the simplest and most stable interaction network with the strongest resistance to the environment and *S. breviflora* had most complex and erratic. Moreover, the bacterial community was mainly affected by dispersal limitation at a certain period. These results are conducive to the prediction of sustainable ecosystem services and protection of microbial resources in a semi-arid grassland ecosystem.

## Introduction

Soil microorganisms play an important role in regulating and maintaining biogeochemical cycles in a terrestrial ecosystem, and are sensitive to environmental changes (Chen et al., 2020). Arid and semi-arid steppe ecosystem, as one of the most important components of the terrestrial system (Kang et al., 2007; Wang et al., 2021), is becoming fragmented and degraded due to climate changes and anthropogenic activity (Gao et al., 2018; Zhang Y. et al., 2018). Climates, vegetation, and soil properties in such steppes vary greatly (Lv et al., 2016), and these changes will strongly affect the structure and function of the soil microbial community (Tu et al., 2017). Numerous studies have focused on soil microbial diversity and assembly processes in arid and semi-arid ecosystems, as well as on the variations and distribution of microorganisms along spatio-temporal, altitude, longitude, plant diversity, and drought gradients (Tu et al., 2017; Yao et al., 2017; Richter-Heitmann et al., 2020; Wang et al., 2021). However, plant root-zone soils have been relatively neglected in complex natural communities. To be specific, the rhizosphere acts as the region of interactions between soil microorganisms and the plant root system's own biological activities (Verma et al., 2019). Moreover, rhizosphere soil microbes are involved in the slow ecological process of vegetation succession and evolution to adapt to the regional environments (Song et al., 2019). This study systematically investigates the turnover and distribution of root-zone soil microbial communities and the correlation with plants along broad spatial gradients of the plant community succession distribution, which is critical to indicating and predicting ecosystem functions.

Abundant rhizosphere microbial resources create a highly evolved external functional environment for plants, thereby allowing organic matters to degrade, nutrients to be released from minerals, nitrogen to be fixed, and elemental forms to be transformed (Lau and Lennon, 2011; Lakshmanan et al., 2014; Na et al., 2018; Krishna et al., 2020). In turn, plants affect microorganisms by the decomposition of litter, turnover of roots, and the release of exudates (Philippot et al., 2013). The interactions help maintain the stability of ecosystem structures and functions, while positively affecting plant fitness and resistance, especially in extreme environments (Marasco et al., 2018). Bacteria comprise a large part of rhizosphere soil biodiversity and participate in most of the material transformation processes (Chu et al., 2020), hence greatly affecting plant growth and establishment. Studies have shown that the size, structure, and activity of the soil bacterial communities could be greatly affected by individual plant species (Liu et al., 2021). Thus, gaining insights into the variation and interactions of plant root-zone soil bacterial communities will enhance our understanding of the ecology and function of soil bacteria. Over the past few decades, spatial and temporal variation in bacterial communities and linkages with plant communities have been extensively investigated in a wide variety of habitats (Tian et al., 2017; Na et al., 2018; Zeng et al., 2019; Chu et al., 2020), whereas such studies have been rare for the root zone of natural forages.

As indicated from existing studies, the soil rhizosphere bacterial composition and relative abundance are highly affected by the biotic and abiotic environments (Fan et al., 2017; Zhang B. et al., 2018). Edaphic factors, particularly soil pH and nutrient availability, vegetation composition and species, regional climate, and altitude, have been shown to shape soil bacterial communities (Na et al., 2018; Zhalnina et al., 2018; Clairmont et al., 2019; Mohanram and Kumar, 2019). Moreover, bacterial evolutionary responses may be driven by edaphic and non-edaphic variables that function as selective pressures; for example, the relationship between the adaptive range of pH and biogeographical patterns (Na et al., 2018), or the greater sensitivity of bacteria in drier areas to environmental stimuli (Maestre et al., 2015). Recently, chemical element indices in soil (e.g., Fe, Cu, Ca, and Mg) have also been proposed to directly impact or predict bacterial diversity and composition (Corneo et al., 2013; Wang et al., 2020). Microbial community assembly (both deterministic and stochastic processes) is critical to maintaining the spatial distribution and composition of microbial communities from a local to a global scale (Stegen et al., 2013; Zhang et al., 2016). Microbial taxa interactions also describe the underlying ecological processes, which can affect the response of communities to environmental variations and may be more important than environmental variables in determining community structures (Zhang B. et al., 2018). For example, it is predicted that ecological networks that consist of weak interactions are more stable than those with strong interactions (Coyte et al., 2015). Yet little is known about how the mentioned ecological processes govern rhizosphere soil bacterial community turnover during the obviously geographic substitution of the plants.

The genus *Stipa* refers to a grazing tolerant, drought-resistant perennial bunchgrass species (Gao et al., 2018; Chen et al., 2020) that is an important ecological barrier and basically in the highly specialized stage of the grassland community succession and evolution (Lv et al., 2016; Liu et al., 2019). From eastern to western Inner Mongolia, across various combinations of hydro-thermal characteristic, different moisture ecotypes of *Stipa* occupy zonal habitats and form meadow steppe, typical steppe, and desert steppe. Accordingly, the *S. grandis* community is generally replaced by the mesophytic *S. baicalensis* community that connects the forest grassland subzone in the east under the progressively humid habitat conditions. As opposed to the mentioned, the *S. grandis* community is largely distributed in the eastern part of the typical steppe, and is often replaced by the *S. krylovii* community when the habitat conditions tend to be dry or frequently grazed. Also, the *S. krylovii* community in the western region of the typical steppe exhibits more xerophytic characteristics and is replaced by the warm-loving *S. breviflora* when the habitat is transmitted to the desert steppe to the west and southwest. Furthermore, *S. breviflora* crosses the southeastern edge of the desert steppe zone from east to west, and then the drier communities of *S. gobica, S. klemenzii*, and *S. glareosa* appear to the northwest. Moreover, *S. gobica* extends westward to the desert, and redundantly is combined with *S. klemenzii* and *S. glareosa* communities (Lv et al., 2016; Li et al., 2018; Nan et al., 2020). *Stipa* taxa show divergences in morphological and physiological parameters (Han and Tian, 2016). The mentioned typical divergent evolution processes provide a unique opportunity and ideal study area to investigate the root-zone bacterial communities in natural ecosystems. The following hypotheses were verified here: (1) There is considerable turnover of root-zone soil bacterial communities along the *Stipa* taxa transition and largely determined by climatic factors, vegetation coverage, and pH at a regional scale. (2) Bacterial communities in root zone have a clear *Stipa* population specificity and the biogeographic distribution pattern would not be affected solely by climatic factors. (3) There would be specific and different bacterial taxa interactions and different resistance to environment in different *Stipa* species, and that the bacterial community assembly is dominated by dispersal limitation. This paper aimed to investigate the geographic distribution, turnover, and interaction of root-zone bacterial community across a transect of zonal *Stipa* species across broad spatial gradients. The identification of potential drivers is critical to elucidating between soil bacterial and plant populations, which can more effectively predict grassland ecosystem responses and functions under a changing climate, and lay a theoretical basis for gaining insights into the community structure and influencing factors of rhizosphere soil bacteria.

## Materials and Methods

### Site Description

**Figure 1:**
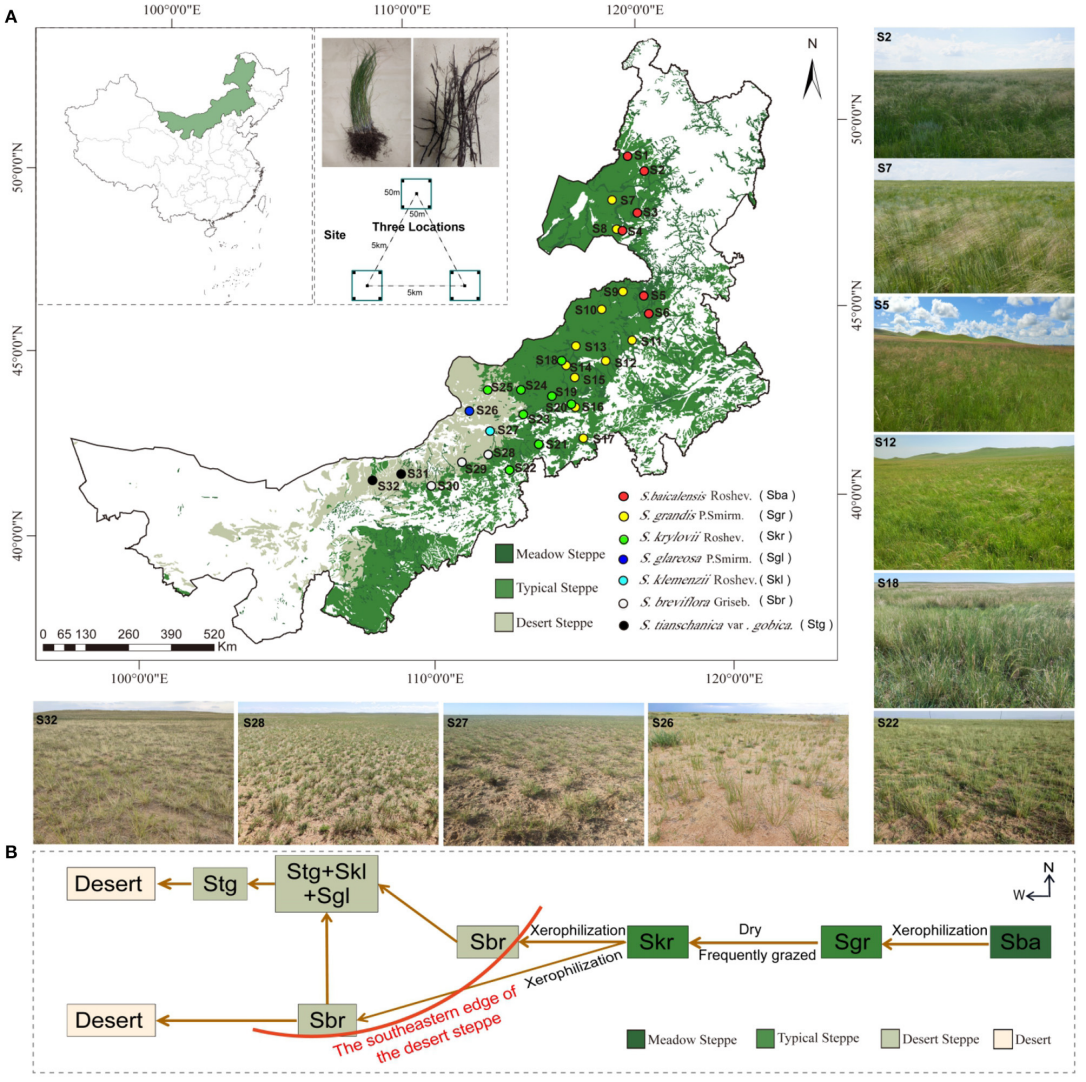
**(A)** Map of sample locations, landscape, and schematic diagram in arid and semi-arid *Stipa* steppe. Each circle represents one sampling site, and each color represents a *Stipa* population; samples collected from different sites and species are referred to as *Stipa baicalensis* Roshev (Sba: S1–S6), *Stipa grandis* P. Smirn (Sgr: S7–S17), *Stipa krylovii* Roshev (Skr: S18–S25), *Stipa glareosa* P. Smirn (Sgl: S26), *Stipa klemenzii* Roshev (Skl: S27), *Stipa breviflora* Griseb (Sbr: S28–S30), and *Stipa tianschanica* var. *gobica* (Stg: S31–S32). **(B)** Schematic diagram of alternative distribution of *Stipa* population.

This study was conducted along a 1,700-km east–west transect across Inner Mongolia (41.52–50.12 N, 108.46–120.21 E) in northern *China*. A total of 32 sampling sites that had nearly natural plant communities with only light animal grazing were selected along the zonal distribution of *Stipa* spp.; six for *S. baicalensis* (S1–S6), 11 for *S. grandis* (S7–S17), eight for *S. krylovii* (S18–S25), three for *S. breviflora* (S28–S30), two for *S. gobica* (S31–S32), and one each for *S. glareosa* (S26) and *S. klemenzii* (S27). Samples were gradually collected from west to east from early August 2019 through the entire transect, to ensure that *Stipa* were in the same mature phenological stage (Figure 1A). Identification of *Stipa* species was according to FLORA INTRAMONGOLICA (EDITIO TERTIA Tomus 6: 205–217). Spatial geographic coordinates, climatic information, and the plant indexes (Plant coverage and Biomass of *Stipa*) for each sampling site are listed in Supplementary Table S2. The region has a temperate continental arid and semi-arid monsoonal climate with a mean annual average precipitation of 172.19–472.06 mm and a mean annual temperature of −0.12–6.56°C (https://disc.gsfc.nasa.gov/). The soil types in the study area include forest grassland black soil and chernozem, chestnut soil in the dry grassland, and brown calcareous soil and lime-calcium soil in the desert steppe.

### Sampling of Root-Zone Soil and Plant Accessions

At the respective site (*n* = 32), three independent replicate locations (50 × 50 m, at least 5 km apart) were selected (Figure 1). In accordance with the double diagonal principle, five 1 × 1 m plots were evenly distributed on the center point of the respective independent location and the two diagonals of the four corners for a vegetation survey, which included plant diversity, number, vegetation coverage, and a calculation of the Shannon–Wiener index ($H^{\prime }=-\overset{r}{\underset{i=1}{\sum }}\left[PiLnPi\right]$, where *Pi* denotes the ratio of the number of each species to the overall number of all species present). The *Stipa* species exhibits a caespitose growth form, which contains numerous tillers and culms emerging from a single rootstock. After establishment, soil, litter, roots, and live shoots accumulate to form tussocks (Bai et al., 1999). The active root system of *Stipa* has rhizosheath (a layer of adhering soil particle to the root surface) that does not easily fall off, creating sufficient water and inorganic salts for the plant and protecting the fibrous roots from mechanical damage (Basirat et al., 2019). The age of perennial bunch grasses is difficult to find through field observation. In the sampling process, plants with tussock fragmentation and self-thinning branches, or too small or excessively large plants should not be selected, as an attempt to collect representative root-zone soil samples and minimize the plant species-age effects on bacterial community diversity and composition (Bai et al., 1999; Na et al., 2018). Given the mentioned information, 25 individual *Stipa* were collected at the respective sampling site, and the excavation depth should exceed 30 cm (Chen, 1987) to ensure the root system to be as complete as possible. To be specific, 15 individual *Stipa* were applied for soil samples and 10 individual *Stipa* for vegetation samples. Several root segments were randomly sampled from each individual *Stipa* after shaking off the extremely loose root-attached soil. Next, the root segments from 15 individual *Stipa* were integrated as one site sample. Subsequently, root-zone soil (Edwards et al., 2015; Shi et al., 2019) was collected from the respective site sample with a disposable brush to brush out the soil around the rhizosheath and then divided equally into three parts. On the whole, 32 × 3 root-zone soil samples were collected, and immediately stored in liquid nitrogen until 16S rRNA high-throughput sequencing analysis could be performed. Brushes and gloves were sterile and disposable to avoid contamination during sampling. The mentioned 10 individual *Stipa* for vegetation samples fell into two equal parts—one part was used for weighing the average aboveground biomass of *Stipa* with the drying method at 80°C, and the other was mixed, crushed, and sieved through a sieve with 0.154 mm pore size into one sample to determine the nutritive value of the forage. The longitude and latitude of each location were recorded from a portable GPS. The collected soil samples fell into two parts: (1) stored at 4°C for soil physical and chemical analysis, and (2) sieved through pore size of 0.074 mm to analyze of soil chemical elements.

### Analysis of Environmental Predictors

A total of 25 environmental predictors were measured. The pH was determined using an SKW 500 soil monitor (Palintest Co., Ltd, UK). Total nitrogen (TN), ammonium (${\text{NH}}_{\text{4}}^{\text{+}}\text{-N}$), nitrate nitrogen (${\text{NO}}_{\text{3}}^{-}\text{-N}$), and plant crude protein (CP) were measured with an automatic Kjeldahl apparatus (K1100 Hanon; Haineng Future Technology Group Co., Ltd, China) after digestion in H_2_SO_4_ (ρ = 1.8419 g/L). Soil organic matter (SOM) and soil organic carbon (SOC) were determined by dichromate oxidation (Chen et al., 2015). Alkaline and neutral Olsen-phosphorus (AP) were determined based on the sodium bicarbonate extraction (0.05 mol·L^−1^) molybdenum antimony anti-colorimetric method (Wang et al., 2020). Available potassium (AK) was determined with a kit according to the instructions (Suzhou Keming Biotechnology Co., Ltd., Suzhou, China). Root-zone soil pretreatment was carried out by microwave digestion system (MARS 6; CEM Co., Ltd, USA) according to a soil and sediment digestion of total metal elements–microwave-assisted acid digestion method (HJ 832−2017, Ministry of Environmental Protection MEP, *China*), and then soil chemical elements (S, Ca, Mg, Fe, Cu, Zn, and Mn) were determined by ICP-OES (PlasmaQuant PQ9000; Jena Analytical Instrument Co., Ltd, Germany). Plant crude fiber and crude fat were measured by an acid–base heating digestion method (GB/T5009.10-2003; Ministry of Health, P. R. China) and a Soxhlet extraction method (GB/T6433-2006; General Administration of Quality Supervision GAQSIQ, China), respectively. Mean annual temperature and mean annual precipitation was obtained from the GES DISC of NASA (https://disc.gsfc.nasa.gov/).

### DNA Extraction, PCR Amplification, and Illumina Sequencing

Triplicate genomic DNA samples were extracted from 0.5 g of the composite soil samples using an E.Z.N.A. soil DNA kit (Omega Bio-tek, Norcross, GA, USA) according to the manufacturer's instructions. The DNA extract was run on a 1% agarose gel, and the DNA concentration and purity were determined with a NanoDrop 2000 UV–vis spectrophotometer (Thermo Scientific, Wilmington, USA). To generate bacterial PCR amplicon libraries, universal 16S rRNA gene primers (338 forward: 5′-ACTCCTACGGGAGGCAGCAG-3′ and 806 reverse: 5′-GGACTACHVGGGTWTCTAAT-3′). The PCR amplification of the 16S rRNA gene was performed according to details in Supplementary Materials. Purified amplicons were pooled in equimolar quantities and paired-end sequencing (2 × 300 bp) on an Illumina MiSeq platform (Illumina, San Diego, USA) according to standard protocols by Majorbio Bio-Pharm Technology Co. Ltd. (Shanghai, China). All of the sequence data generated for this study have been deposited in the NCBI Sequence Read Archive (SRR14415681–SRR14415776).

### Bioinformatics Workflow

The raw 16S rRNA gene sequences obtained from the MiSeq platform were quality filtered, trimmed, de-noised, and merged using Mothur 1.32.2 (Schloss et al., 2009) where chimeric sequences were identified and removed using the UCHIME *de novo* algorithm (Perez-Jaramillo et al., 2019). Subsequently, the remaining high-quality sequences were clustered into operational taxonomic units (OTUs) using the UPARSE (version 7.0.1090, http://www.drive5.com/uparse/) algorithm, setting a distance limit of 0.03 using the open-reference OTU picking protocol. A representative sequence was aligned using the RDP Classifier (http://rdp.cme.msu.edu/) against the 16S rRNA database (Silva 132; https://www.arb-silva.de/) using a confidence threshold of 0.7. For downstream analysis, OTUs affiliated with chloroplasts and mitochondria were subsequently removed from the bacterial OTU table, and OTUs that were assigned to non-bacteria, including plants and protozoans, were removed from the bacterial OTU table (Perez-Jaramillo et al., 2019). The effects of sampling on diversity were corrected by rarifying the sequence numbers of each sample to that of the sample with the lowest number of reads (17,865 reads). The number of OTUs, community richness (Chao index), community diversity (Simpson index), community evenness (Shannon index), and a sequencing depth index (Good's coverage) were subsequently calculated using MOTHUR (v.1.30.1; www.mothur.org).

### Statistical Analysis

All statistical analyses were conducted through a one-way ANOVA with IBM SPSS Statistics 26.0 (*p* < 0.05 was considered statistically significant). Non-metric multidimensional scaling [NMDS, stress < 0.20 was considered acceptable (Kuczynski et al., 2011)] and hierarchical clustering (unweighted pair group method with arithmetic means, UPGMA) that all used the Bray–Curtis distance to assess the *Stipa* population-specificity distribution of bacteria, as well as the similarity of bacterial community composition. To demonstrate the effect of *Stipa* population on the bacterial composition, permutational multivariate analysis of variance (PERMANOVA, *p* < 0.05) was conducted with the adonis function of the vegan package (Glasl et al., 2019) with 999 permutations. A linear discriminant analysis (LDA score threshold of 4.0) coupled with effect size (LEfSe) was adopted to search for statistically different biomarkers among groups of *Stipa* taxa root zone. In addition, Circos-0.67-7 was employed to determine the connection between the dominant phylum or genus and the sample group of *Stipa* species. The variance inflation factor (VIF) was exploited to remove the redundant variables (VIF > 20) (Fan and Xing, 2016) from the explanatory variables and avoid collinearity among a range of factors. The Mantel test and the Pearson correlation analysis were performed to examine the relationship between bacterial communities and environmental factors using the “Vegan” package (Oksanen et al., 2015) and the “ggcor” package (Wang et al., 2021) version 0.9 implemented in R, respectively. A redundancy analysis (RDA) was also performed to determine the most significant environmental variables shaping the microbial community composition.

Bacterial co-occurrence network analyses for *Stipa* species were conducted based on the Molecular Ecological Network Analyses Pipeline (MENAP, http://ieg4.rccc.ou.edu/MENA/main.cgi) at the OTU level (Deng et al., 2012). To simplify the networks for a more effective visualization and unified analysis conditions, only the 300 most abundant OTUs were analyzed and network topology parameters (i.e., network complexity and modularity) were determined to indicate the stability of the network and the resistance from environmental interference. Abundances of OTUs were log transformed. To compare networks under the identical conditions, a threshold of 0.82 (the recommended similarity threshold) was adopted to build the networks for the respective *Stipa* taxa in the study. Lastly, the networks were visualized with the Gephi 0.9.2 (Zhou et al., 2020). Furthermore, 100 random networks were generated and the properties were compared with the experience network to verify whether the constructed network is reasonable and effective. The different assembly processes of community composition were determined by phylogenetic null model analyses. All OTUs were adopted to build a maximum-likelihood tree in FastTree (Tang et al., 2021) to determine the weighted β-nearest taxon index (βNTI) using the picante package (Dini-Andreote et al., 2015). The βNTI values > +2 (variable selection) or < −2 (homogeneous selection) implies significantly more or less phylogenetic turnover than expected (Tripathi et al., 2018), respectively, thereby demonstrating the predominance of deterministic processes. If the |βNTI| ≤ 2, stochastic processes are predominant. To more specifically differentiate between stochastic scenarios of assemblages of *Stipa* species root-zone soil bacteria, a Raup–Crick matrix (RCbray) based on the Bray–Curtis matrix was computed with the vegan package (Cheng et al., 2021). |βNTI| < 2 and RCBray < −0.95, |βNTI| < 2 and RCBray > 0.95, and |βNTI| < 2 and |RCBray| < 0.95 denote homogenizing dispersal, dispersal limitation, and undominated processes, respectively (e.g., weak selection, weak dispersal, diversification, and/or drift) (Stegen et al., 2013; Dini-Andreote et al., 2015). Furthermore, unless otherwise indicated, all analyses were conducted in *R* (version 3.5.1; *R* Development Core Team).

## Results

### Vegetation and Soil Characteristics of the Study Area

In accordance with Supplementary Tables S1, S2, the MAP of the semi-arid grassland decreased gradually from east (472.06 mm) to west (152.08 mm). SOM, SOC, TN, ${\text{NH}}_{4}^{+}$-N, VC, and SB tended to increase with precipitation, while pH, altitude, and temperature tended to decrease. The soils were moderately alkaline and the average pH increased significantly from 6.06 to 8.74. Other test indices displayed significant differences among 32 sites, but no obvious rules to follow. There were significant differences in vegetation indexes among seven *Stipa* species, and the vegetation coverage (66.67 ± 14.38 to 27.50 ± 3.61) and *Stipa* biomass (3.00 ± 1.31 to 0.85 ± 0.32) decreased significantly under the process of Sbr replacing Sba distribution (Supplementary Table S3). Most soil parameters differed significantly between the various root-zone soils of *Stipa*, except for TK and ${\text{NO}}_{3}^{\text{-}}$-N, in which macronutrients (SOM, SOC, TN, ${\text{NH}}_{4}^{+}$-N) in Sba were significantly higher than for other *Stipa* taxa (Supplementary Table S3). A Pearson correlation analysis (**Figure 6**) found that most variables (MAP, MAT, altitude, soil pH, SOC, TN, S, VS, and SB) had strong positive or negative correlations with each other. In addition, there was a significant positive correlation between soil chemical elements.

### Turnover of Root-Zone Bacterial Community Composition of *Stipa* Taxa

We obtained a total of 4,665,705 raw reads by Illumina MiSeq high-throughput sequencing. After quality filtering, trimming, and assigning reads to the different samples, 3,150,865 high-quality reads were recovered in the dataset, representing 17,865 bacterial operational taxonomic units (OTUs) based on 97% sequence identity across all samples, with a median of 2,349 OTUs per sample (Supplementary Table S4). The observed OTUs and rarefaction curves of Shannon index, Chao richness, and Good's coverage (values were over 0.94) in all soil samples were plateaued (Supplementary Figure S1), suggesting that the sequencing depth was sufficient.

**Figure 2:**
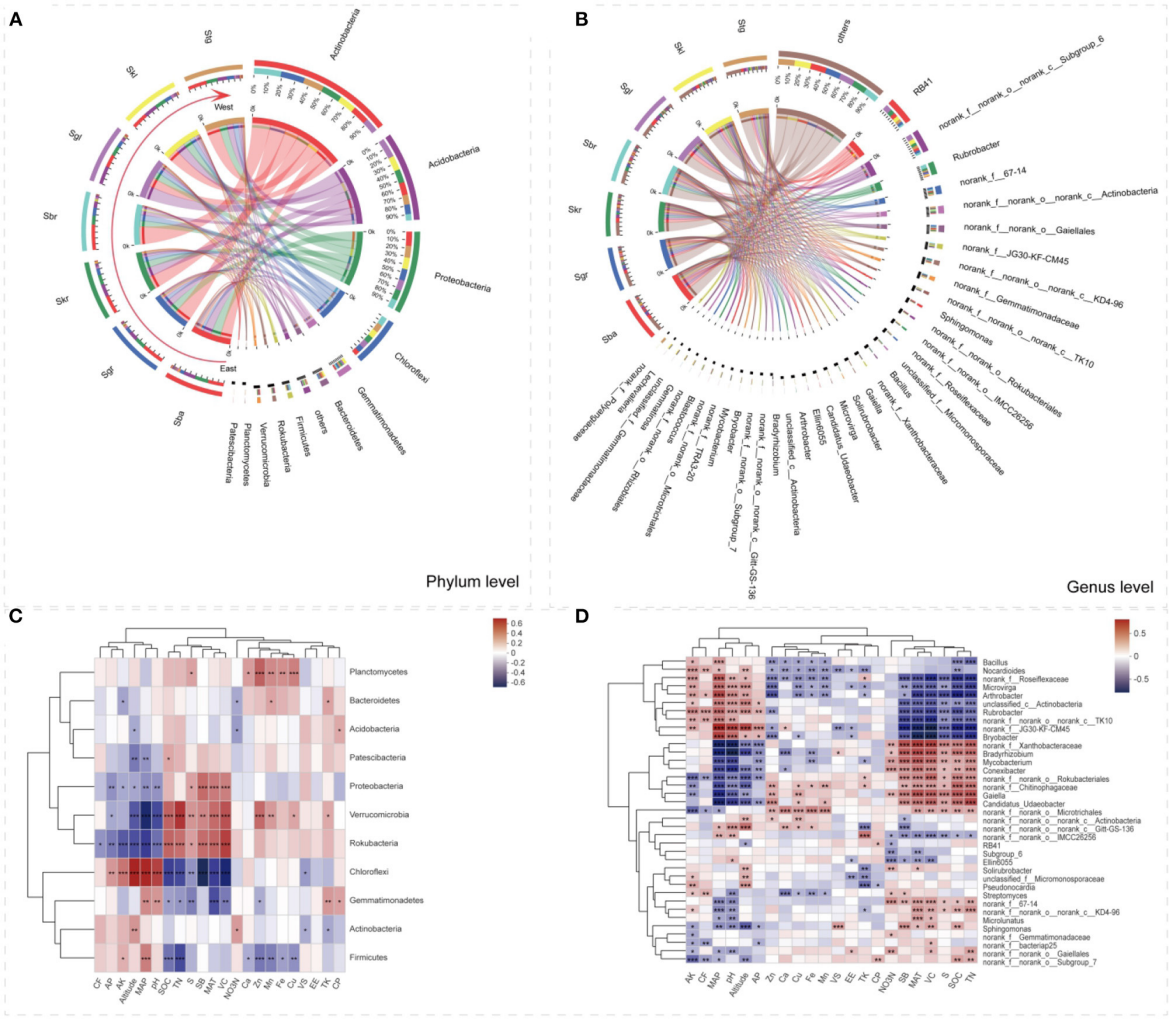
The distributions of root-zone soil bacterial dominant taxa at the different *Stipa* species. **(A)** Represents the phylum level; **(B)** represents the genus level, and Spearman correlation analysis of relative abundance of bacterial keystone taxa with environmental factors: **(C)** phylum level, **(D)** genus level. Red represents positive correlation and blue represents negative correlation (significance levels are as follows: *0.01 < *p* ≤ 0.05, **0.001 < *p* ≤ 0.01, ***p ≤ 0.001). Arrows refer to the geographic substitution order of *Stipa* species from east to west. SOC, soil organic carbon; TN, total nitrogen; TK, total kalium; ${\text{NO}}_{3}^{\text{-}}$-N, nitrate nitrogen; AP, alkaline and neutral Olsen-phosphorus; AK, available potassium; Fe, iron element; Cu, copper element; Zn, zinc element; Mn, manganese element; Ca, calcium element; S, sulfur element; VS, Shannon–Wiener index of plot; VC, plant coverage; SB, biomass of *Stipa*; CP, crude protein; EE, crude fat; CF, crude fiber; MAT, mean annual temperature; MAP, mean annual precipitation. *S. baicalensis* (Sba), *S. grandis* (Sgr), *S. krylovii* (Skr), *S. glareosa* (Sgl), *S. klemenzii* (Skl), *S. breviflora* (Sbr), and *S. gobica* (Stg).

Circular visualization diagrams were adopted to display the overlapping and differentiating bacterial taxa among the seven *Stipa* taxa at the phylum and genus levels (Figures 2A,B). The bacterial community consisted of 37 different phyla, and the main bacterial phyla (top 11) collectively accounted for 96.98–98.76% of all taxon sequences (Figure 2A). Moreover, the five most abundant bacterial genera comprised 27.71% of the bacterial communities on average (Figure 2B). *Actinobacteria, Acidobacteria, Proteobacteria*, and *Chloroflexi* were the most abundant phyla observed in all of the *Stipa* taxa analyzed (Figure 2A). Except for Sgl, *Actinobacteria* (28.34–45.35%) was the dominant phylum in other *Stipa* species groups, especially in Sbr root zone (45.35%). *Acidobacteria* had a most significant advantage as dominant phylum in Sgl (32.81%) compared with other six *Stipa* taxa (10.73–24.10%). When Sba was replaced by Sbr from east to west along the transect, the relative abundance of *Chloroflexi* (phylum, from 8.17 to 14.80%) and *Rubrobacter* (genus, from 1.89 to 10.20%) increased significantly, and *Rubrobacter* was the most dominant genus in Sbr (10.20%); as opposed to the mentioned, the relative abundances of *Proteobacteria* (from 24.06 to 15.70%) decreased significantly (Figures 2A,B and Supplementary Table S6). Except Sbr, the relative abundances of genus *RB41* (7–15.27%) and *Subgroup_6* (5.85–13.04%) in the root zone of *Stipa* taxa were significantly higher than for other genera, especially in Sgl (Figure 2B). Taken together, these results revealed shifts in the bacterial community composition in root-zone soils of different *Stipa* taxa.

**Figure 3:**
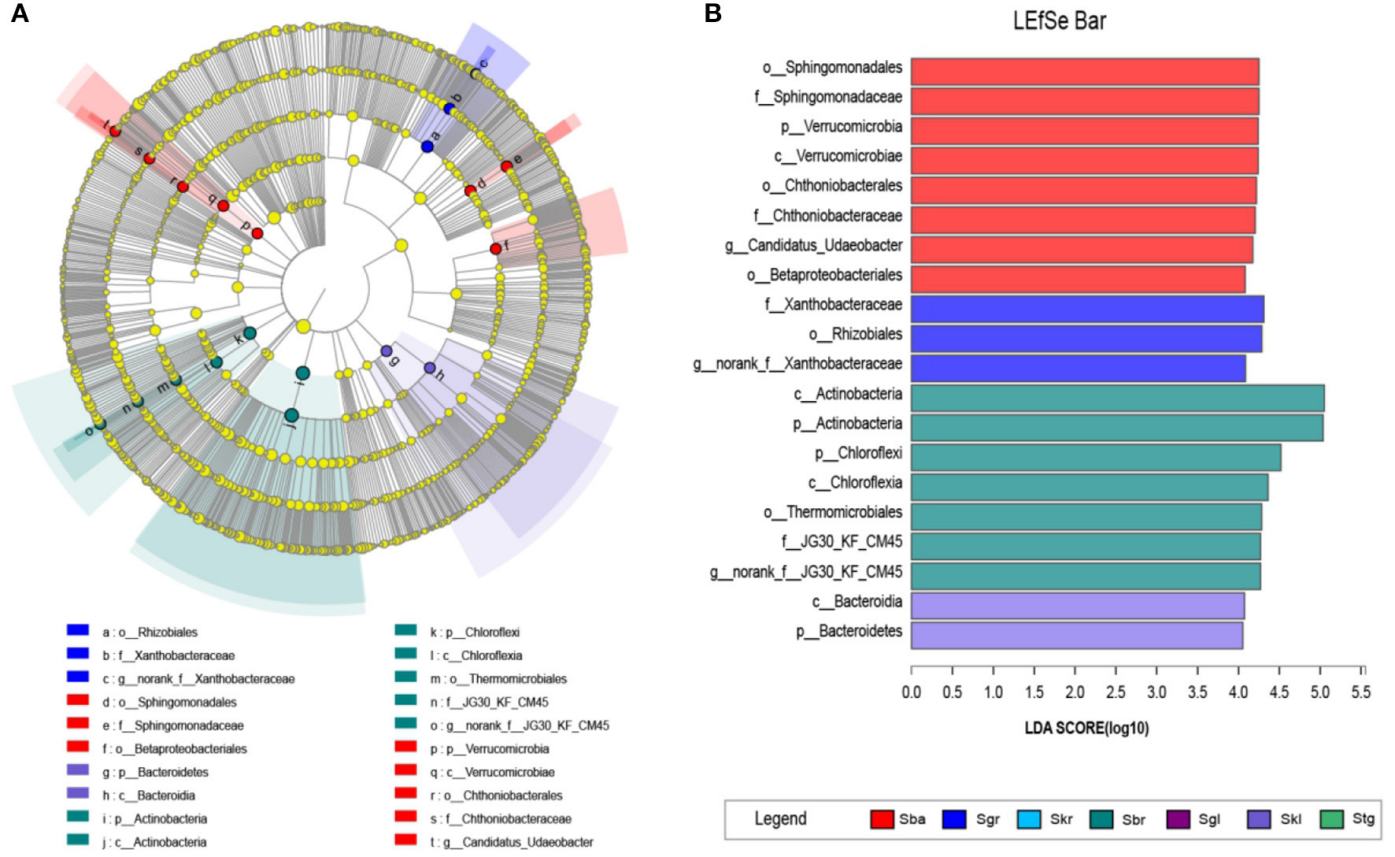
LEfSe analysis of soil bacterial abundance in seven *Stipa* species root-zone soils. **(A)** Cladogram of microbial communities. Cladograms indicate the phylogenetic distribution of microbial lineages associated with the study *Stipa*; circles represent phylogenetic levels from kingdom to genus. **(B)** Indicator microbial groups with LDA scores > 4 and *p*-values < 0.05. *S. baicalensis* (Sba), *S. grandis* (Sgr), *S. krylovii* (Skr), *S. glareosa* (Sgl), *S. klemenzii* (Skl), *S. breviflora* (Sbr), and *S. gobica* (Stg).

The LEfSe with LDA scores of 4 (Figure 3) revealed that, when compared with the Sba root-zone soil bacterial community (eight biomarkers at finer taxonomic level, one phylum, one class, three orders, two families, and one genera), the other *Stipa* species root-zone soil communities had less biomarkers (Figure 3A). However, Skr, as an intermediate transitional species, had no biomarkers. Furthermore, Stg and Sgl were distributed over a small area and had no biomarkers. To be specific, root-zone soil of Skl showed two significantly different taxa, *Bacteroidia* (class) and *Bacteroidetes* (phylum); Sgr were rich in *Rhizobiales* (order), *Xanthobacteraceae* (family), and *norank_f__Xanthobacteraceae* (genus); Sba were enriched with *Sphingomonadales* (order), *Chthoniobacterales* (order), *Betaproteobacteriales* (order), *Sphingomonadaceae* (family), *Chthoniobacteraceae* (family), and *Candidatus_Udaeobacter* (genus), while the root-zone soil of Sbr was enriched with *Thermomicrobiales* (order), *JG30_KF_CM45* (family and genus) (Figure 3B).

### Bacterial Community Diversity Varied and Spatial Distribution of *Stipa* Taxa Root-Zone Soils

**Figure 4:**
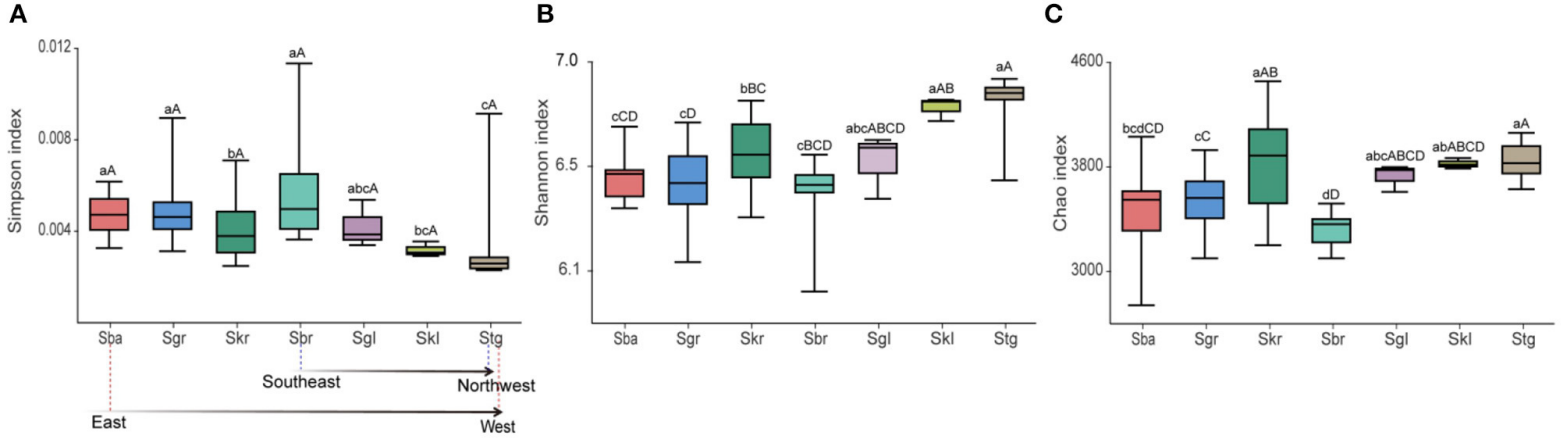
Alpha-diversity indices of bacterial communities in root-zone soils of seven *Stipa* species along a transect from east to west. Different letters (a–g, *p* < 0.05/A–D, *p* < 0.01) above the bars show significant difference among groups based on one-way ANOVA. **(A)** Simpson index expresses microbial diversity. **(B)** Shannon index expresses microbial evenness. **(C)** Chao index expresses microbial richness. *S. baicalensis* (Sba), *S. grandis* (Sgr), *S. krylovii* (Skr), *S. glareosa* (Sgl), *S. klemenzii* (Skl), *S. breviflora* (Sbr), and *S. gobica* (Stg).

The bacterial biodiversity (Simpson index, Figure 4A) of the root-zone soil decreased when Sba was replaced by Skr from southeast to northwest along the transect, increased for Sbr, and then decreased sharply as Sbr transitioned to Stg. The trends of indices richness (Chao index Figure 4C) and evenness (Shannon index Figure 4B) of bacterial were opposite with the variation patterns of the mentioned bacterial diversity. We found that the α diversity of bacterial communities was different across 32 sampling sites with respect to the bacterial richness, diversity, and evenness for 96 soil samples, and a lower bacterial diversity was observed in more arid areas, as indicated by the changes in the Simpson index (Supplementary Table S2). Furthermore, bacterial evenness (Shannon index) and diversity (Simpson index) were more affected by altitude and pH, and bacterial evenness also affected crude protein; bacterial richness, as indicated by the Chao index, was affected mostly by the altitude and was also principally connected with crude fat (**Figure 6**).

**Figure 5:**
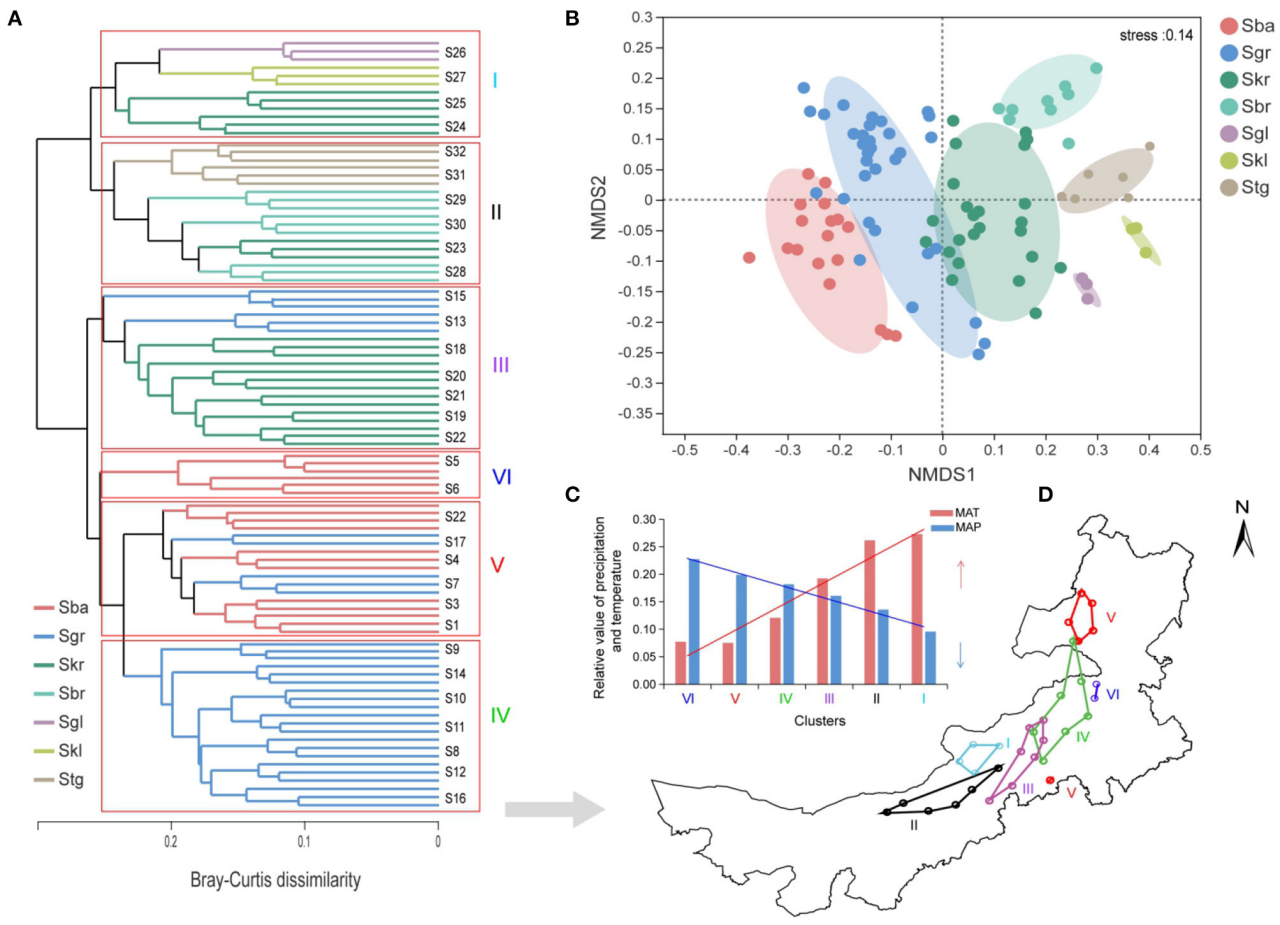
Bacterial community differentiation in the root-zone soils from seven *Stipa* species sampled from different sites of arid and semi-arid steppe. **(A)** Similarity analysis of bacterial community in *Stipa* taxa root zone based on UPGMA clustering using Bray–Curtis distances, clustered into six clusters (I–VI), roman numeral color corresponds to **(C)**. **(B)** Population specificity of *Stipa* root-zone soil bacterial communities. **(C)** Columnar trend graphs of relative mean annual precipitation (MAP) and temperature (MAT) in different clusters; roman numerals correspond to **(A,D)**. **(D)** Biogeographical patterns of bacterial communities, with same color clustering. *S. baicalensis* (Sba), *S. grandis* (Sgr), *S. krylovii* (Skr), *S. glareosa* (Sgl), *S. klemenzii* (Skl), *S. breviflora* (Sbr), and *S. gobica* (Stg).

NMDS analysis revealed a clear separation of the bacterial communities from different *Stipa* taxa (Figure 5B), and *Stipa* population specificity was further confirmed with a PERMANOVA (*p* = 0.001, Supplementary Table S5). In addition, the UPGMA clustering analysis also revealed similarity distribution in the bacterial community composition based on sample sites (Figure 5A). The samples formed two clear large clusters; moreover, six clear small clusters (I–VI) were generated, and two of these included only from Sba (VI) and Sgr (IV). The bacterial community was roughly distributed from southeast (VI) to northwest (I) along the reduced precipitation gradient and increased temperature gradient (Figures 5C,D). MAP (*R*^2^ = 0.78, *p* < 0.01) was more dominant than the *Stipa* taxa (*R*^2^ = 0.71, *p* < 0.01) for the distribution of bacterial community, which was indicated by the NMDS plot ordination analysis (Supplementary Table S7). In addition, soil pH (*R*^2^ = 0.63, *p* < 0.01), vegetation coverage (*R*^2^ = 0.64, *p* < 0.01), and MAT (*R*^2^ = 0.68, *p* < 0.01) also had a strong effect on the distribution of the bacterial communities across the geographical replacement gradient of the *Stipa* taxa (Supplementary Table S7). The mentioned results indicate that the composition of root-zone bacterial community not only had the *Stipa* population specificity, but also was strongly regulated by climate, and had a change pattern along the hydrothermal gradient. Especially in the process of transition from Sba to Sgr and then to Skr, root-zone bacteria distribution has a certain phenomenon of cross overlap.

### Ecological Factors Effecting Root-Zone Bacterial Community Spatial Turnover

**Figure 6:**
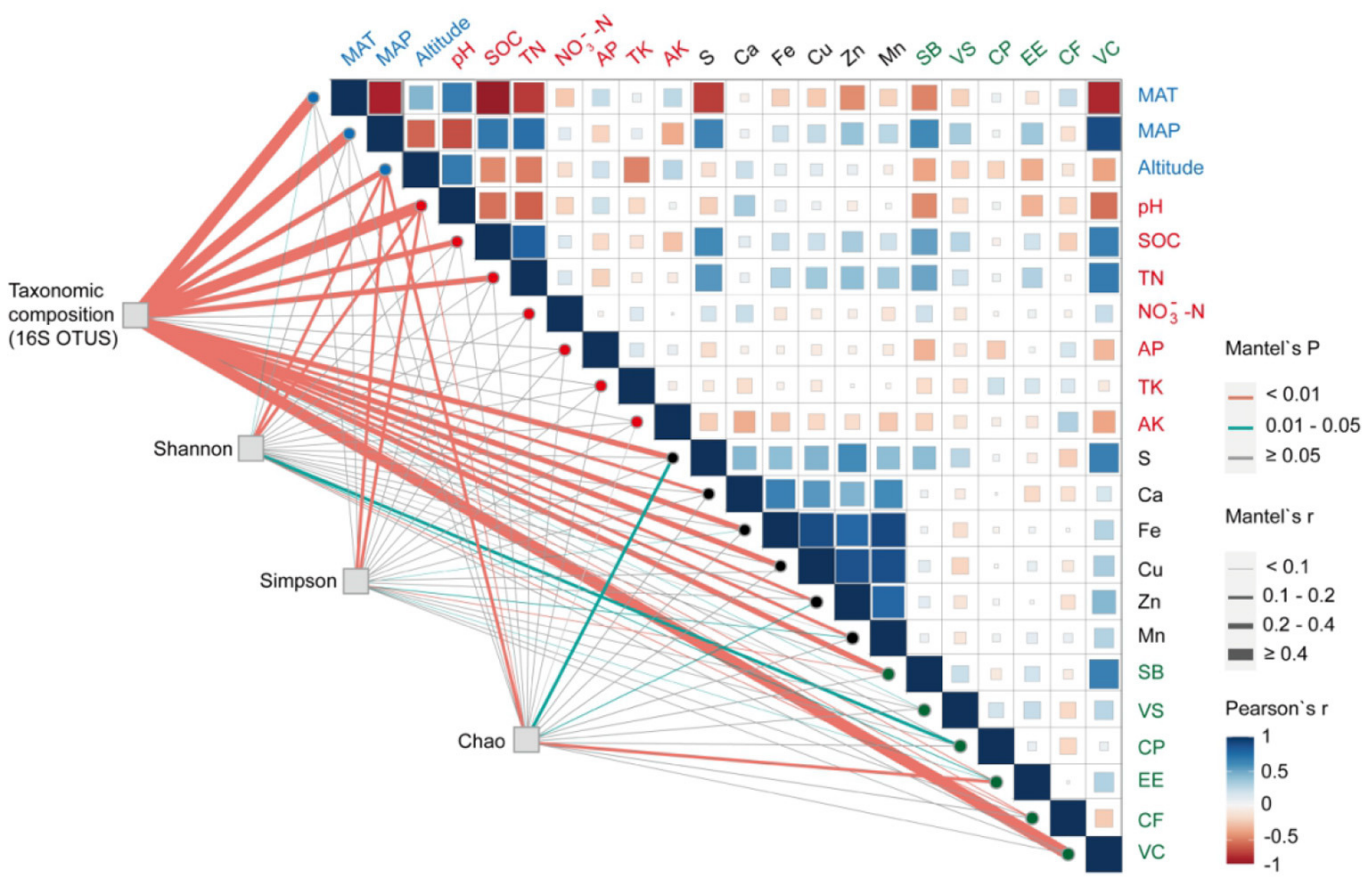
Mantel correlations among bacterial community composition (OTU level), bacterial richness (Chao index), bacterial diversity (Simpson index), bacterial evenness (Shannon index), and environmental factors (climate, plant, and soil variables); Pearson correlation analysis was used among environmental factors. The width of edges represents the size of the correlation coefficient (Mantel's r), while edge color represents the statistical significance based on Mantel's *p*. Abbreviations are consistent with Figure 2.

Three redundant variables (${\text{NH}}_{4}^{+}$-N, SOM, and Mg) were eliminated based on VIF analysis to reveal the major environmental variables shaping bacterial community composition and turnover. RDA and Mantel test were conducted to distinguish the impact of soil properties, climate, and vegetation factors on bacterial communities. RDA1 and RDA2 axis, respectively, explained 22.54 and 9.97% of the variance in soil bacterial community structures (Supplementary Figure S2A). The Mantel tests (Figure 6) denoted that the compositions of soil bacterial communities were significantly constructed by MAP (r = 0.58), MAT (r = 0.65), pH (r = 0.50), and VC (r = 0.53). These results were also supported by the RDA (Supplementary Figure S2A). The soil chemical elements (i.e., S, Fe, and Cu) also had an effect on the bacterial community structure, but not on the bacterial community distribution (Figure 6, Supplementary Figure S2A, and Supplementary Table S7). SOC, TN, and SB were significantly related to the spatial turnover and distribution of the root-zone soil bacterial community (Figure 6, Supplementary Figure S2A, and Supplementary Table S7). In accordance with the RDA examined, the bacteria phyla at seven *Stipa* root zones were mainly effected by VC (conditional effect = *R*^2^ = 0.26, *p* = 0.001), SB (*R*^2^ = 0.22, *p* = 0.001), and MAT (*R*^2^ = 0.19, *p* = 0.001; Supplementary Figure S2B). At genus level, MAP (conditional effect = *R*^2^ = 0.60, *p* =0.001), MAT (*R*^2^ = 0.52, *p* = 0.001), MAT (*R*^2^ = 0.51, *p* = 0.001), pH (*R*^2^ = 0.41, *p* = 0.001), SB (*R*^2^ = 0.39, *p* = 0.001), SOC (*R*^2^ = 0.38, *p* = 0.001), and TN (*R*^2^ = 0.38, *p* = 0.001) affected bacteria, and all bacteria with relative abundance >1% could be explained by one soil property at least (Supplementary Figure S2C). The correlation heatmap denoted the relationship between relative abundance of bacterial keystone taxa with environmental factor (Figures 2C,D). It was found that the relative abundances of most dominant bacterial phyla and genus were significantly related to most soil physicochemical properties (i.e., soil pH, TN, AP, AK, S, Zn, Mn, Fe, Cu, and SOC), plant factors (i.e., SB and VC), and other indicators (i.e., MAT, MAP, and altitude). Furthermore, *Firmicutes* was significantly positively correlated with soil chemical elements (except S); on the contrary, *Planctomycete*s was significantly negatively correlated.

### Bacterial Taxa Interactions and Community Assembly Processes in the Root Zone of *Stipa*

**Figure 7:**
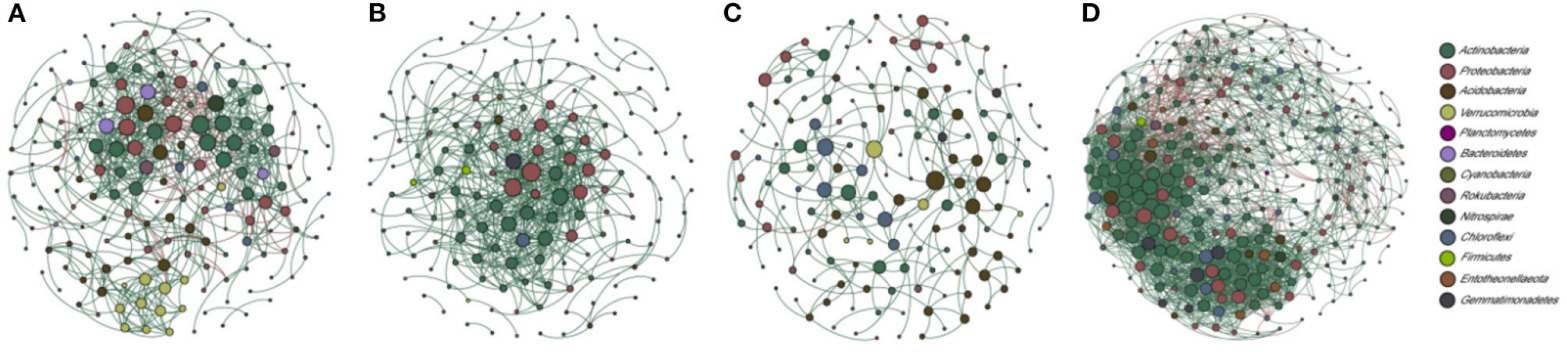
Co-occurrence networks of the high-abundant bacteria from four *Stipa* species root-zone soil; in turn is *S. baicalensis* (Sba: **A**), *S. grandis* (Sgr: **B**), *S. krylovii* (Skr: **C**), and *S. breviflora* (Sbr: **D**). A connection stands for a robust (Spearman's r > |0.82|) and significant (*p* < 0.05) correlation. Green and red lines represent positive and negative associations between two nodes, respectively. Nodes are colored according to microbial phylum and the size of each node is proportional to the degree.

To explore the complex bacterial taxa interactions in different *Stipa* taxa root-zone soils and to identify the response to environmental change, a co-occurrence network analysis for abundant OTUs (top 300 OTUs) was performed. The Sgl, Stg, and Skl root-zone soils were excluded from the analyses because of limited samples. Based on the number of links (Sba = 648, Sgr = 527), avgK (Sba = 7.16, Sgr = 6.47), and the avgCC (Sba = 0.38, Sgr = 0.33), the network complexity was comparable between the Sba and Sgr root-zone soils, with nodes in the control network, respectively, grouped into 21 and 25 modules with respective quality values of 0.59 and 0.38 (Figures 7A,B and Supplementary Table S8). Moreover, the complexity (links = 226, avgK = 2.83, avgCC = 0.32) was less in Skr root-zone soils (Figure 7C), and the network grouped into 22 (quality value of 0.81) modules. However, the complexity (links = 2593, avgK = 18.13, avgCC = 0.49) greatly increased in Sbr root-zone soils (Figure 7D), which contained six modules, with a quality value of 0.49. There were absolutely more positive correlations in each network than negative correlations. *R* square of power-law values of Sba, Sgr, and Skr networks were obtained for 0.87, 0.78, and 0.76, respectively, indicating the occurrence of scale-free network characteristics (Supplementary Table S8).

The βNTI values were determined, to report that the stochastic process was dominant in the bacterial communities of seven *Stipa* species root-zone soils (Supplementary Figure S3A; values >-2 and < 2). We calculated the taxonomic-diversity metric RCBray to further quantify the relative effects of various ecological processes, including homogenizing dispersal, dispersal limitation and undominated processes, on the stochastic community assembly of every group (Supplementary Figure S3B). Consequently, the relative effects of dispersal limitation (24–49%) and undominated processes (27–41%) increased significantly across the transition from Sba to Skr, while the relative role of homogenizing dispersal (10–48%) was significantly reduced. In arid areas, the relative role of homogenizing dispersal (33–100%) was dominant in the bacterial community assembly in the root zone of *Stipa* species, except for Stg. Overall, the relative effects of dispersal limitation (73%) of *Stipa* genus root-zone soil bacteria were the most important in the grassland system.

## Discussion

### Turnover of the Root-Zone Bacterial Communities Along the *Stipa* Taxa Transition

Diverse bacterial composition and structure were detected in root-zone soils when the *Stipa* taxa replace each other in space. According to the dominant bacteria taxon, *Actinobacteria* achieved the maximal relative abundance in most *Stipa* species root-zone soils (except for Sgl), which might be attributed to the climate and the soil characteristics of the research area. The members of the bacterial phylum *Actinobacteria* have been proven as an indicator of drought sensitivity and drought tolerance, and exhibit a strong metabolic capacity at low temperatures (Cheng et al., 2021). Given the Circos analysis, the top two *Stipa* taxa in *Acidobacteria* group were Sgl and Skl, which were located in the desert steppe that was characterized by low soil nutrition; in addition, *Acidobacteria* was the most dominant phylum in the Sgl root-zone soil; *Acidobacteria* was composed of various oligotrophs (Zeng et al., 2019), capable of tolerating harsh conditions (Yao et al., 2017). The phylum *Proteobacteria* comprises diverse groups of copiotrophic organisms in soil normally identified under nutrient-rich conditions (Janssen, 2006). The nutrient richness of root-zone soil of Sba is much higher than for other *Stipa* taxa (Supplementary Table S3), and the relative abundance of the phylum *Proteobacteria* decreased significantly in the process of Sbr replacing Sba. Moreover, the phylum *Proteobacteria* was negatively correlated with MAT, pH, and AP. The relative abundance of *Proteobacteria* in Sbr root zone was lower than that of several *Stipa* taxa (Sgl, Skl, and Stg) with higher content of AP. Root-zone bacterial phyla and genera of the *Stipa* varied significantly following the vegetation replacement distribution gradient (Supplementary Table S6). For instance, when Sba was replaced by Sbr from east to west following the transect, the relative abundance of the phylum *Chloroflexi* and the genus *Rubrobacter* was improved significantly, and *Rubrobacter* was reported as the most dominant genus in Sbr root-zone soil (Supplementary Table S6 and Figure 2). The phylum *Chloroflexi* pertains to the group of heterotrophic oligotrophs in soil (Hug et al., 2013), and the genus *Rubrobacter* is significantly radiation resistant, halotolerant, thermotolerant, or even thermophilic (Kourilova et al., 2021). Most bacteria exhibited a relatively narrow growth tolerance, thereby causing individual taxa to be unable to adapt to soil pH variations (Fierer and Jackson, 2006), and then probably inducing variations of soil bacterial communities (Shen et al., 2013). pH (near neutral range) is suggested to exert a mediating effect in grassland system (Tripathi et al., 2018). This may explain why the relative abundance of *Actinobacteria* and *Acidobacteria* in this paper was considerable, whereas this has no significant correlation with neutral soil pH, while the areas exhibiting an acidic soil pH showed a significant correlation [e.g., the bacterial community in *Changbai* Mountain (Han et al., 2018)]. Together with the significant correlations between soil pH and SOC, TN and ${\text{NH}}_{4}^{+}$-N of root-zone process of grassland might explain why soil pH affected the root-zone bacterial community by affecting the availability of soil carbon and nitrogen or the number of biogeochemical processes involving carbon and nitrogen cycles (Jiao and Lu, 2020), thereby complying consistent with the research on *Caragana* spp. rhizosphere soil (Na et al., 2018). All the mentioned results collectively suggested that the variations of whole bacterial communities are strongly ecological-variable dependent and various phyla and genus may show different variation patterns.

### Biomarkers of Soil Bacteria in the Root Zone of *Stipa* Taxa

Besides the differences in the relative abundance of the identical bacterial taxa, soils in different *Stipa* root zones also harbor different potential biomarkers (Figure 3), which have been characteristically isolated from soil samples or plant root systems. The Skl only had two significant taxa, *Bacteroidia* (class) and *Bacteroidetes* (phylum), that were involved in C and N metabolism and occurring extensively across various ecological niches (Wang et al., 2020). Several species of the family *Sphingomonadaceae* (*Sphingomonadales*) utilize a wide range of organic compounds for growth and survival under low-nutrient conditions (Glaeser and Kämpfer, 2014). The family *Chthoniobacteraceae* (*Chthoniobacterales*), represented by the genera *Chthoniobacter* and *Candidatus Xiphinematobacter*, can metabolize organic carbon (Victoria et al., 2012) and prevent nutrient leaching and erosion (Kant et al., 2011). The order *Betaproteobacteriales* exhibited a large relative abundance in these bacterial community studies. However, it is significantly enriched in the Sba than other *Stipa* taxa. Given the vulnerability of several species pertaining to *Betaproteobacteriales* to salinity, acidity, and grazing pressure, Sba achieves a better growth and living environment than other *Stipa* taxa. Moreover, Sba itself is easily replaced in heavily grazing and salinized areas. Several species of the order *Thermomicrobiales* (the family and genus of *JG30_KF_CM45*) are characterized as thermophilic, neutral, and basophilic (*Bergey's Manual of Systematics of Archaea and Bacteria*). *Norank_f__JG30_KF_CM45* acts as a potential genus biomarker for the Sbr, with a significant positive correlation with MAT, pH, and AP, but a negative correlation with MAP, SOC, TN, and S (Figure 2B), while *Candidatus_Udaeobacter* (Sba) and *norank_f__Xanthobacteraceae* (Sgr) exhibit opposite behaviors (Figure 2B). The mentioned explains why the warm-loving, drought-resistant, alkali-resistant, and barren-resistant *norank_f__JG30_KF_CM45* is enriched, which may indicate available phosphorus transformation and utilization. The critical thing is that Sbr also likes warmth (Chen et al., 2020). *Candidatus_Udaeobacter* and *norank_f__Xanthobacteraceae* act as potential biomarkers for the Sba and Sgr, respectively, which might indicate vital ecosystem multifunctionality involved in SOC accumulation, S cycle, and N stock across the chronosequence (Li et al., 2021). The family *Xanthobacteraceae* (*Rhizobiales* and *norank_f__Xanthobacteraceae*) in the Sgr grows with aerobic chemoheterotrophs and fix nitrogen (Oren, 2014; Yu et al., 2019). The mentioned functional bacteria in root zone can directly indicate the soil nutrient transformation and the response of bacterial to environmental variations.

### Climate Is the Main Driving Factor for the Turnover and Distribution of Bacterial Community in the Root Zone

Climate has been recognized as a vital driver of soil bacterial community structure in regional and large-scale studies (Zeng et al., 2019). Yao proved climate drives the differentiation of bulk soil bacterial communities in the eastern Inner Mongolia steppe (Yao et al., 2017). Likewise, temperature and precipitation are two major climatic factors affecting the *Stipa* species root-zone soil bacterial community structure and distribution in this paper. This is related to the influence of precipitation on plant community composition and soil nutrient availability. For instance, higher precipitation may upregulate soil organic matter decomposition rate, thereby reducing soil organic matter availability (Tian et al., 2017; Na et al., 2019), and increasing microbial metabolic rates and biochemical processes due to temperature (Zhou et al., 2016), which subsequently affects the soil bacterial community structure. Furthermore, the spatial pattern of root-zone soil bacterial communities was distributed along the hydrothermal combination gradient. The mentioned result was attributed to the long-term influence of the southeast ocean monsoon and the northeast–southwest arc mountain barriers (Greater *Khingan* Mountains and the *Yin* Mountains), thereby causing the climatic factors to form an arc belt distribution. Such a peculiar water–heat combination causes the zonation of soil and vegetation, which makes the differentiation of vegetation zones in this area roughly coincide with the distribution of the climatic zones (Han and Tian, 2016).

Plant and soil parameters were other crucial factors of the bacterial community structure and distribution. As indicated from existing studies, soil pH exerts a dominant effect on various ecosystems in shaping bacterial structures and large-scale spatial distributions (Fierer and Jackson, 2006; Fan et al., 2017), mainly in acidic and neutral soil environments (Yang et al., 2018). Different soil properties play a leading role in an alkaline environment (Wang et al., 2017); for example, the soil carbon content was the dominant factor of bacteria distribution in the *Ali* area (Chu et al., 2016). The result of this paper partially complies with the existing studies indicating that soil pH was the most crucial soil parameter, whereas SOC, SOM, TN, and ${\text{NH}}_{4}^{+}$-N significantly affected the structure and distribution of *Stipa* taxa root-zone soil bacterial communities. In this paper, chemical element (S, Fe, and Cu) also impacted the bacterial community structure, whereas it did not impact the bacterial community distribution except for S (Figure 6 and Supplementary Table S7). Thus, the soil sulfur cycle plays a partial role in the ecological distribution of bacteria, whereas microbes in rhizosphere are critical to allowing plants to access soil organosulfur (Kertesz et al., 2007). This paper also reported that the plant coverage and biomass of *Stipa* had a significant impact on the distribution and structure of the bacterial community at the phylum and genus levels were particularly prominent (Supplementary Figures S2, S6); complying with Han's view (Han et al., 2018), while the species of *Stipa* significantly impacted the distribution of bacteria. This is because a higher plant biomass and differences in the plant cover are generally directly affecting the quality and quantity of organic matter input, with subsequent effects on bacterial communities (Fierer and Jackson, 2006). In arid systems, the changes in plant coverage are also especially important for nutrient and moisture maintenance (Yao et al., 2017). Furthermore, different vegetation types can determine litter composition, soil conditions, and resource acquisition strategies, thereby indirectly regulating the structure of the bacterial community through a superposition effect with soil factors (Yao et al., 2018). In contrast, the soil microbial community structure will also affect the natural selection pattern of plant traits, while regulating the response of plants to abiotic environmental pressure, thereby affecting the evolutionary process of the whole ecosystem (Berg and Smalla, 2009). Besides, high interspecific clustering of the bacterial root-zone communities was identified. Such an interspecific difference could be partially determined by the genetic diversity among host species (Yu and Hochholdinger, 2018).

On the whole, the effect of precipitation and *Stipa* species on the structure and distribution of the root-zone soil bacterial community exceeded that of soil pH on a large scale. It is noteworthy that the bacterial community composition in the root zone of *Stipa* taxa complied with the previous reports of bulk or rhizosphere soil in a semi-arid system (Wang et al., 2015; Nan et al., 2020), while the bacteria community structure was different. Many studies have confirmed that the rhizosphere microbiome consists of a subset of the bulk soil microbiome; besides, microbial community diversity decreased rapidly from bulk soil to rhizosphere (Reinhold-Hurek et al., 2015; Trivedi et al., 2020). During the assembly of microbial communities from bulk soil to rhizosphere, the bulk soil provides a microbial seed bank, the physical–chemical properties, the biogeography, and climate conditions (Zhang et al., 2017). Based on reasons discussed previously, the differences observed in the root zone of *Stipa* also might be mediated by the bulk soil microbiota in their respective regions.

### Soil pH and Altitude Have Strong Correlation With α Bacterial Community Diversity

The diversity, evenness, and richness of rhizosphere bacteria were inconsistent in the seven plant species. There was a sudden change in the root-zone soil bacterial diversity at Sbr in desert steppe. According to Dijkstra et al. (2012), bacterial diversity changed suddenly in arid areas, and soil microbes living in zones with scarce water availability might be easily activated by even small rainfall events, which did not reach a level that would satisfy the plants' needs. As reported from numerous studies, soil pH acts as a vital factor driving the diversity of rhizosphere soil bacterial community (Na et al., 2018; Wang et al., 2020). This paper further examined the previously found strong correlation between soil pH and bacterial root-zone community diversity (Figure 6), as the pH conditions affect the adaptation and selection of particular phylogenic groups (Wang et al., 2020). In turn, bacterial α diversity, as indicated by the Shannon and Chao indexes, significantly affects the nutrient composition of forage grasses, especially the accumulation of crude fat and crude protein, whereas the correlation coefficient is relatively small. In addition, the elevation is correlated with the variables (SOC, TN, TK, and pH) affecting the ecosystem and plays a key role in influencing root-zone bacterial diversity, evenness, and richness. Wang reported that microbial richness in the Qinghai–Tibet Plateau showed a significant negative correlation with altitude. The pattern of microbial diversity and altitude was inconsistent due to the differences in the primary factors regulating the bacterial diversity in soils from different regions (Wang et al., 2020). Furthermore, this paper reported that root-zone soil bacterial α diversity was positively correlated with altitude.

### Bacterial Taxa Interaction and Community Assembly in *Stipa* Root-Zone Soil

Both deterministic and stochastic processes were governing the spatial distribution of microbial communities concurrently, and different processes were found to be dominant in various cases (Zhang et al., 2016). The root-zone bacterial community assembly was dominated by stochastic processes at *Stipa* taxa, which also complied with the results of several studies, i.e., the effects of stochastic processes were commonly dominant at larger geographic scales (Li et al., 2021). Previous research has shown that the spatio-temporal distribution of soil bacteria in a temperate grassland was dominated by stochastic dispersal (Richter-Heitmann et al., 2020). According to other studies, the variation of microbial community structure will be dominated by different assembly factors in different periods (Zhou et al., 2014; Liu et al., 2020). The results of this paper may only represent the assembly of *Stipa* rhizosphere microorganisms in such a period, which can be exploited to determine the stability of different *Stipa* rhizosphere bacterial communities of the period (Zhou and Ning, 2017). The significant variations of drought span and soil structure in the study area may impose the natural limitation on the undirected dispersal of microbes like others have reported (Li and Hu, 2021), which also explains why the relative effects of dispersal limitation of *Stipa* genus root-zone soil bacteria showed the absolute advantage in the study. As expected, the relative effects of dispersal limitation and undominated processes increased significantly across the transition from Sba to Skr, while the relative role of homogenizing dispersal decreased significantly (Supplementary Figure S3B), thereby demonstrating that the bacterial community structure tended to be increasingly stable, and environmental variations exerted fewer effects (Zhou and Ning, 2017). Homogeneous selection was the main process driving the assembly of Sbr, Sgl, and Skl bacterial communities, showing that community structure would change largely with environmental conditions (Cheng et al., 2021).

This paper reported that the potential correlation patterns of different *Stipa* taxa root-zone bacterial groups varied substantially for network complexity and modularity. Moreover, the M, GD, and avgCC of the empirical networks were clearly higher than those of the respective random networks (Supplementary Table S8), suggesting that empirical networks had a noticeable hierarchy and modularity in their topological properties. Some studies have reported that the interactions of root-associated microbes are more complex than those of microbes in bulk soil, which may be related to rhizosphere microbial diversity, and the main interactions are positive (>80%), which also indicates that rhizosphere may have greater potential for cooperative or mutualistic associations (Coyte et al., 2015; Fan et al., 2018), and so are the root zones of *Stipa*, because *four* networks had a large proportion of members that are connected through positive links (Supplementary Table S8). However, such bacterial community is considered unstable since bacterial taxa may make a coincident response to environmental fluctuations, thereby inducing positive feedback and co-oscillation (Coyte et al., 2015). According to the bacterial co-occurrence pattern characterized by a high average degree, an average clustering coefficient, and fewer modules and modularity at extremely arid areas indicated that the networks of Sbr root-zone soil exhibit a more complex bacterial community structure with closer and better-connected nodes, whereas such topological features are of low stability (De Vries et al., 2018). Nevertheless, the Skr network was simplest but most stable based on topological characteristics. This network exhibited stronger resistance to environmental variations. Skr is easy to replace other *Stipa* taxa when the growth environment is stressed, thereby also indicating that the population can strongly adapt to the environment. This also reveals that there may be potential co-evolution relationship between vegetation and their root-zone bacteria. In summary, network analysis and RCbray results exert a good indication and prediction effect on environmental interference. More network complexity and fewer modules were reported in the severe environment than in the mild environment, thereby demonstrating that network complexity of bacteria was facilitated by various environmental factors, whereas the clustering of modules was not.

## Data Availability Statement

The original contributions presented in the study are included in the article/Supplementary Materials, further inquiries can be directed to the corresponding author.

## Author Contributions

XM: investigation, data curation, methodology, visualization, writing—original draft, writing—review, and editing. LC, SW, and JL: investigation. ZD and JL: data curation. FL: field investigation. YB: funding acquisition, supervision, resources, review, and editing. All authors contributed to the article and approved the submitted version.

## Funding

This study was supported by the National Natural Science Foundation of China (31760005) and Science and Technology Major Project of Inner Mongolia Autonomous Region of China (Grant No. zdzx2018065).

## Conflict of Interest

The authors declare that the research was conducted in the absence of any commercial or financial relationships that could be construed as a potential conflict of interest.

## Publisher's Note

All claims expressed in this article are solely those of the authors and do not necessarily represent those of their affiliated organizations, or those of the publisher, the editors and the reviewers. Any product that may be evaluated in this article, or claim that may be made by its manufacturer, is not guaranteed or endorsed by the publisher.
