# Supplementary material for: The Distribution and Turnover of Bacterial Communities in the Root Zone of Seven Stipa Species Across an Arid and Semi-arid Steppe
Source: Front Microbiol. 2021 Dec 24;12:782621. doi: 10.3389/fmicb.2021.782621 (PMC8741278; doi:10.3389/fmicb.2021.782621)
Supplement: Supplementary file 1 [file Data_Sheet_1.docx]

Supplementary Material

1. **DNA extraction, PCR amplification and purification**

Microbial community genomic DNA was extracted from *Stipa* root-zone soil samples (0.5g/per) using the E.Z.N.A.® soil DNA Kit (Omega Bio-tek, Norcross, GA, U.S.) according to manufacturer’s instructions. The DNA extract was checked on 1% agarose gel, voltage 5 V/cm for 20min and DNA concentration and purity were determined with NanoDrop 2000 UV-vis spectrophotometer (Thermo Scientific, Wilmington, USA). The hypervariable region V3-V4 of the bacterial 16S rRNA gene were amplified with primer pairs 338F (5'-ACTCCTACGGGAGGCAGCAG-3') and 806R(5'-GGACTACHVGGGTWTCTAAT-3') by an ABI GeneAmp® 9700 PCR thermocycler (ABI, CA, USA). The PCR amplification of 16S rRNA gene was performed as follows: initial denaturation at 95 ℃ for 3 min, followed by 27 cycles of denaturing at 95 ℃ for 30 s, annealing at 55 ℃ for 30 s and extension at 72 ℃ for 45 s, and single extension at 72 ℃ for 10 min, and end at 10 ℃. The PCR mixtures contain 5 × TransStart FastPfu buffer 4 μL, 2.5 mM dNTPs 2 μL , forward primer (5 μM) 0.8 μL, reverse primer (5 μM) 0.8 μL, TransStart FastPfu DNA Polymerase 0.4 μL, BSA 0.2 μL,template DNA 10 ng, and finally ddH2O up to 20 μL. PCR reactions were performed in triplicate. The PCR product was extracted from 2% agarose gel and purified using the AxyPrep DNA Gel Extraction Kit (Axygen Biosciences, Union City, CA, USA) according to manufacturer’s instructions and quantified using Quantus™ Fluorometer (Promega, USA). In addition, sterile water was used as negative control for DNA extraction and amplification.

**Table S1** **|** Site geographic information and selected meteorological and spatial parameters used for this study.

| *Stipa* taxa | Sample | Sampling region | Latitude/longitude coordinates (D) | Altitude (m) | MAT(℃) | MAP(mm) |
| --- | --- | --- | --- | --- | --- | --- |
| Sba | S1 | Ergun Banner | E119.6157 / N50.1194 | 525.9 | 1.15 | 442.35 |
|  | S2 | Hailar | E120.2124 / N49.6320 | 673.9 | -0.12 | 448.30 |
|  | S3 | Yimin Sumu | E119.6691 / N48.4934 | 745 | 1.30 | 406.83 |
|  | S4 | Xin Barag Left Banner | E118.9482 / N48.0936 | 761 | 1.62 | 362.97 |
|  | S5 | East Ujimqin Banner | E119.3330 / N46.1402 | 987.7 | 1.04 | 472.06 |
|  | S6 | East Ujimqin Banner | E119.5269 / N45.7584 | 930.4 | 2.24 | 452.74 |
| Sgr | S7 | Prairie Chenbarhu banner | E118.8338 / N48.8809 | 717.5 | 1.53 | 336.83 |
|  | S8 | Xin Barag Left Banner | E118.8734 / N48.1163 | 752 | 1.69 | 354.82 |
|  | S9 | East Ujimqin Banner | E118.7262 / N46.3169 | 875.8 | 2.17 | 426.05 |
|  | S10 | East Ujimqin Banner | E117.7865 / N45.9150 | 948.7 | 2.09 | 329.03 |
|  | S11 | West Ujimqin banner | E118.8480 / N44.9533 | 984.9 | 2.12 | 402.51 |
|  | S12 | West Ujimqin banner | E117.7260 / N44.4660 | 1163.2 | 3.15 | 406.26 |
|  | S13 | East Ujimqin Banner | E116.6281 / N44.9774 | 851 | 3.98 | 320.84 |
|  | S14 | xilin hot | E115.9876 / N44.4933 | 989.7 | 4.66 | 308.75 |
|  | S15 | xilin hot | E116.4467 / N44.1004 | 1098 | 3.79 | 324.89 |
|  | S16 | xilin hot | E116.1660 / N43.4287 | 1458.1 | 2.06 | 360.59 |
|  | S17 | Zhenglan Banner | E116.5212 / N42.4600 | 1263 | 4.08 | 430.50 |
| Skr | S18 | xilin hot | E115.9614 / N44.6105 | 1016.5 | 4.32 | 300.67 |
|  | S19 | Abag Banner | E115.4065 / N43.7963 | 1125.6 | 4.23 | 302.11 |
|  | S20 | xilin hot | E116.0853 / N43.5012 | 1378 | 3.06 | 340.00 |
|  | S21 | Zhengxiangbai Banner | E114.7686 / N42.4920 | 1295.8 | 4.90 | 327.04 |
|  | S22 | Chahar Right Back Banner | E113.5821 / N41.9165 | 1466 | 4.32 | 375.64 |
|  | S23 | Abag Banner | E114.2866 / N43.3581 | 1081.5 | 5.37 | 234.39 |
|  | S24 | Abag Banner | E114.3127 / N44.0816 | 1058 | 4.78 | 233.11 |
|  | S25 | Sonid Left Banner | E113.0181 / N44.0591 | 980.3 | 5.59 | 172.19 |
| Sgl | S26 | Erenhot | E112.2074 / N43.5742 | 960.5 | 6.25 | 152.08 |
| Skl | S27 | Sonid Right Banner | E112.9513 / N42.9628 | 1076 | 6.56 | 220.81 |
| Sbr | S28 | Sonid Right Banner | E112.8079 / N42.3102 | 1208 | 6.18 | 254.66 |
|  | S29 | Siziwang Banner | E111.9378 / N41.7702 | 1439.3 | 4.98 | 331.14 |
|  | S30 | Baotou | E110.6313 / N41.5279 | 1445 | 5.63 | 355.48 |
| Stg | S31 | Baotou | E109.5182 / N41.9235 | 1498.5 | 5.50 | 248.98 |
|  | S32 | Urad Front Banner | E108.4610 / N41.7851 | 1531 | 5.65 | 232.31 |

Notes: *S. baicalensis* (Sba), *S. grandis* (Sgr), *S. krylovii* (Skr), *S. glareosa* (Sgl), *S. klemenzii* (Skl), *S. breviflora* (Sbr), and *S. gobica* (Stg).MAT: mean annual temperature; MAP: mean annual precipitation.

**Table S3** **|** Plant variables of seven different *Stipa* taxa and physical and chemical characteristics of root-zone soil.

|  | **Index** | **Sba** | **Sgr** | **Skr** | **Sbr** | **Sgl** | **Skl** | **Stg** |
| --- | --- | --- | --- | --- | --- | --- | --- | --- |
| plant variables | VC (%) | 66.67±14.38a | 47.42±12.26b | 31.88±11.11bc | 27.50±3.61cd | 14±0.50de | 10.00±1.73e | 16.67±0.00cde |
|  | SB (g/per) | 3.00±1.31a | 2.78±0.87a | 1.25±0.69b | 0.85±0.32b | 0.96±0.01b | 0.72±0.07b | 0.30±0.01b |
|  | VS (%) | 2.25±0.41a | 1.86±0.41a | 2.02±0.37a | 2.06±0.46a | 2.26±0.46a | 1.74±0.39a | 1.08±0.77b |
|  | CP (%) | 1.03±0.06ab | 0.99±0.046bc | 1.06±0.07ab | 1.02±0.06ab | 1.08±0.01a | 1.06±0.02ab | 0.91±0.10c |
|  | EE (%) | 0.63±0.02a | 0.62±0.03abc | 0.61±0.04abc | 0.62±0.03abc | 0.62±0.01ab | 0.59±0.02bc | 0.58±0.03c |
|  | CF (%) | 1.41±0.02ab | 1.43±0.03ab | 1.41±0.02ab | 1.43±0.03ab | 1.43±0.02a | 1.40±0.02b | 1.44±0.02a |
| Soil physics | pH | 6.63±0.41d | 6.97±0.57cd | 7.60±0.48bc | 7.98±0.073ab | 7.99±0.01ab | 8.21±0.05ab | 8.31±0.47a |
|  | SOM (g/kg) | 32.58±4.74a | 19.50±5.42b | 18.29±4.53bc | 12.19±1.31cd | 7.86±0.87d | 12.02±0.10cd | 9.38±1.58d |
|  | SOC (g/kg） | 20.17±2.91a | 11.77±3.78b | 10.31±3.48bc | 7.07±2.57cd | 4.94±0.30d | 5.50±0.06d | 6.30±0.83cd |
|  | TN (g/kg) | 1.94±0.80a | 1.45±0.70ab | 0.92±0.48bc | 0.48±0.25c | 0.17±0.02c | 0.22±0.01c | 0.29±0.02c |
|  | NH_4_^+^-N (μg/g) | 33.52±10.73a | 26.43±9.280ab | 19.23±6.03bc | 13.84±3.40c | 9.44±0.08c | 10.41±0.10c | 11.04±0.35c |
|  | NO_3_^-^-N (μg/g) | 1.83±2.39a | 2.12±2.16a | 1.43±1.29a | 0.80±0.21a | 0.36±0.01a | 0.54±0.44a | 0.68±0.29a |
|  | AP (μg/g) | 16.57±8.70b | 18.77±8.57ab | 18.78±9.21ab | 28.13±6.66a | 22.23±3.29ab | 25.58±3.55ab | 24.89±5.76ab |
|  | TK (mg/L) | 155.66±10.25a | 148.86±14.18a | 148.98±15.42a | 139.40±10.12a | 147.30±6.25a | 155.40±2.60a | 157.40±5.70a |
|  | AK (g/kg) | 55.57±18.16b | 73.11±11.32ab | 82.28±16.01a | 69.52±15.05ab | 77.42±0.70a | 69.85±0.76ab | 63.16±1.51ab |
| Soil chemistry | Mg (mg/L) | 27.28±6.88bc | 21.83±6.58c | 23.84±7.90c | 34.15±4.65ab | 8.57±0.10d | 17.97±0.08c | 38.59±4.63a |
|  | Fe (mg/L) | 93.285±18.093ab | 74.78±17.77bc | 75.21±20.73bc | 98.13±11.08ab | 29.89±1.05d | 54.32±0.62cd | 108.80±7.67a |
|  | Cu (mg/L) | 0.120±0.023ab | 0.101±0.017bc | 0.093±0.024bc | 0.12±0.021ab | 0.062±0.007d | 0.078±0.002cd | 0.132±0.021a |
|  | Zn (mg/L) | 0.228±0.057ab | 0.152±0.055abc | 0.143±0.108bc | 0.166±0.059abc | 0.013±0.000d | 0.077±0.001cd | 0.247±0.096a |
|  | Mn (mg/L) | 2.600±0.631a | 1.969±0.556abc | 1.842±0.548bcd | 2.528±0.300ab | 1.388±0.100cd | 1.187±0.0130d | 2.638±0.356a |
|  | S (mg/L) | 3.97±0.74a | 2.43±0.87b | 2.28±1.33b | 2.30±0.87b | 0.92±0.04c | 1.30±0.02bc | 1.79±0.23bc |
|  | Ca (mg/L) | 62.23±27.70ab | 56.04±44.27ab | 58.67±37.39ab | 88.23±45.92a | 18.57±1.42b | 42.41±0.30ab | 64.53±1.73ab |

Note: Values for soil chemical properties within the same row followed by different letters indicate significant differences (P < 0.05, ANOVA, Tukey’s HSD test). SOC: soil organic carbon; TN: total nitrogen; TK: total kalium; NO_3_^-^-N: nitrate nitrogen; NH_4_^+^-N: ammonium; AP: alkaline and neutral olsen-phosphorus; AK: available potassium; Fe: iron element; Cu: copper element; Zn: zinc element; Mn:manganese element; Ca: calcium element; S: sulfur element; VS: Shannon-Wiener index of plant; VC: plant coverage; SB: biomass of *Stipa*; CP: crude protein; EE: crude fat; CF: crude fibre. *S.baicalensis* (Sba), *S. grandis* (Sgr), *S. krylovii* (Skr), *S. glareosa (*Sgl), *S. klemenzii* (Skl), *S. breviflora* (Sbr), and *S. gobica* (Stg).

**Table S5 |** Statistical output of PERMANOVA (adonis, vegan package) testing the effect of *Stipa* population on the bacterial composition (999 permutations, Bonferroni adjusted p values).

| Characteristics | Df | SumsOfSqs | MeanSqs | F_Model | R^2^ | *P*_value | *P*_adjust |
| --- | --- | --- | --- | --- | --- | --- | --- |
| *Stipa* | 7 | 5.455056267 | 0.909176045 | 9.587016675 | 0.392583099 | 0.001 | 0.001 |
| Residuals | 89 | 8.440234403 | 0.094834094 | - | 0.607416901 | - |  |
| Total | 96 | 13.89529067 | - | - | 1 | - |  |

**Table S7 |** Effects of plant characteristics, climate factors and soil physicochemical properties on the geographical distribution of bacterial communities based on the NMDS plot ordination of the Bray-Curtis distances, R^2^ is the coefficient of determination, representing the proportion of variation explained by the linear regression.

| Influential factors | | Bacteria | |
| --- | --- | --- | --- |
|  |  | *R^2^* | *P* |
| Climatic factors | **MAP** | **0.78** | 0 |
|  | **MAT** | **0.68** | 0 |
| Soil physics and chemistry | **pH** | **0.63** | 0 |
|  | **SOM** | **0.44** | 0 |
|  | **SOC** | **0.43** | 0 |
|  | **TN** | **0.45** | 0 |
|  | **NH_4_^+^-N** | **0.45** | 0 |
|  | NO_3_--N | 0.05 | 0.03 |
|  | AP | 0.11 | 0 |
|  | AK | 0.05 | 0.03 |
|  | TK | 0 | 0.57 |
|  | Ca | 0.03 | 0.11 |
|  | S | 0.26 | 0 |
|  | Mg | 0.04 | 0.04 |
|  | Fe | 0 | 0.91 |
|  | Cu | 0 | 0.51 |
|  | Zn | 0.04 | 0.05 |
|  | Mn | 0.01 | 0.37 |
| Plant variables | **SB** | **0.43** | 0 |
|  | **VC** | **0.64** | 0 |
|  | VS | 0.07 | 0.01 |
|  | EE | 0.06 | 0.02 |
|  | CF | 0.01 | 0.48 |
|  | CP | 0 | 0.6 |
|  | ***Stipa* taxa** | **0.71** | 0 |
|  | Alititude | 0.35 | 0 |

Notes SOC: soil organic carbon; SOM:soil organic matter; TN: total nitrogen; TK: total kalium; NO_3_^-^-N: nitrate nitrogen; NH_4_^+^-N: ammonium; AP: alkaline and neutral olsen-phosphorus; AK: available potassium; Fe: iron element; Cu: copper element; Zn: zinc element; Mn:manganese element; Ca: calcium element; S：sulfur element; Mg:Magnesium element; VS：Shannon-Wiener index of plot; VC: plant coverage; SB: biomass of *Stipa*; CP: crude protein; EE: crude fat; CF：crude fibre; MAT: mean annual temperature; MAP: mean annual precipitation.

**Table S8** **|** Topological features of networks in the different *Stipa* species root-zone soil associated high-abundant bacterial communities (Top 300 of total abundance) across all sites based on the Spearman correlation method.

|  | **Empirical Network Indexes** | | | | | | | | | **Random networks** | | |
| --- | --- | --- | --- | --- | --- | --- | --- | --- | --- | --- | --- | --- |
| taxa | Total nodes | Total links | R square of power-law | Positive | Average degree (avgK) | Transitivity | Average path distance (GD) | Average clustering coefficient (avgCC) | Modularity(M) | Average path distance (GD) | Average clustering coefficient (avgCC) | Modularity(M) |
|  |  |  |  | /negative edges |  |  |  |  |  |  |  |  |
| **Sba** | 181 | 648 | 0.87 | 0.88/0.13 | 7.16 | 0.483 | 3.99 | 0.374 | 0.59(21) | 2.925±0.043 | 0.086 ±0.010 | 0.304 ± 0.007 |
| **Sgr** | 163 | 527 | 0.78 | 1/0 | 6.47 | 0.428 | 3.061 | 0.325 | 0.38(25) | 2.942 ± 0.053 | 0.134 ± 0.013 | 0.302± 0.007 |
| **Skr** | 160 | 226 | 0.76 | 0.99/0.01 | 2.83 | 0.36 | 6.377 | 0.32 | 0.81(22) | 4.807±0.106 | 0.015±0.008 | 0.621±0.011 |
| **Sbr** | 286 | 2593 | 0.51 | 0.89/0.11 | 18.13 | 0.549 | 3.43 | 0.486 | 0.49(6) | 2.402±0.014 | 0.141±0.006 | 0.171±0.004 |

Notes:*S.baicalensis* (Sba), *S. grandis* (Sgr), *S. krylovii* (Skr) and *S. breviflora* (Sbr).


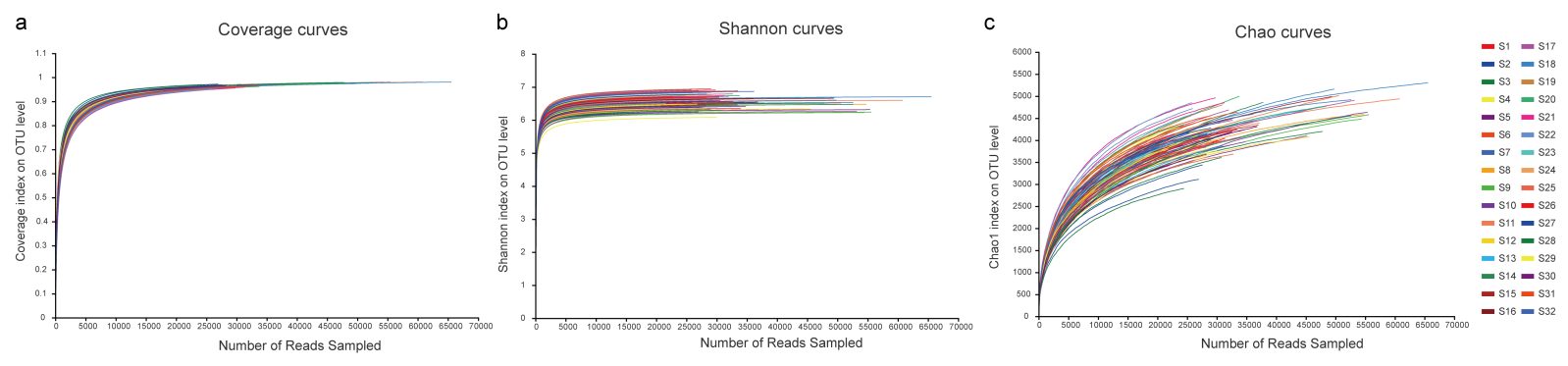


**Figure S1** | The rarefaction curves of (a) Good’s coverage, (b) Shannon index, and (c) Chao index at OTU level for 32 sites (96 samples).


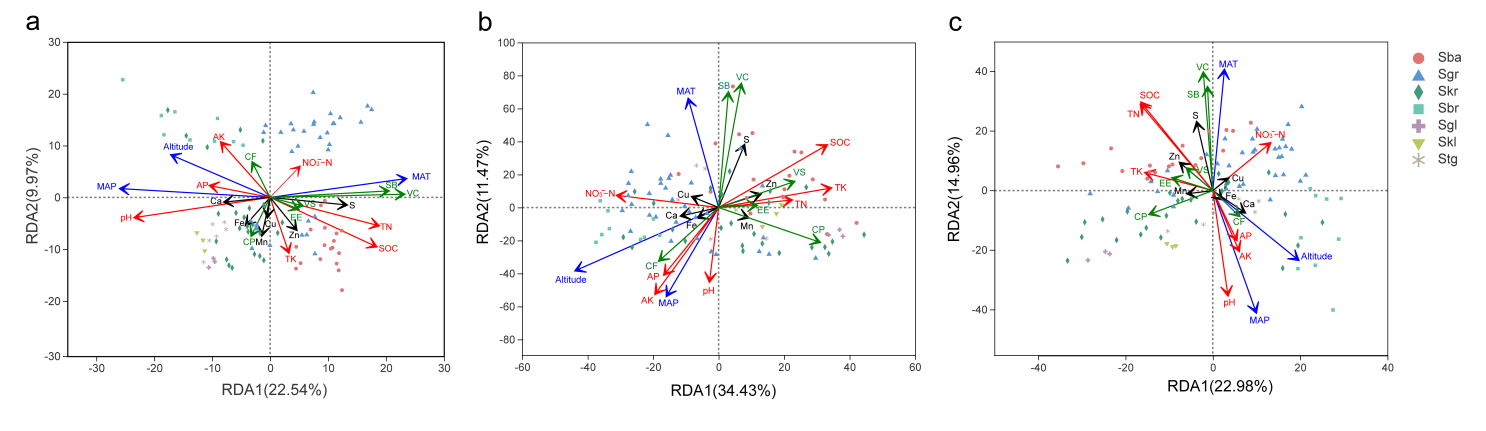


**Figure S2 |** Relationships between bacterial community structure and environmental factors used redundancy analysis (RDA). **a)** bacterial OTU level; **b)** bacterial phyla level; **c)** bacterial genera level. The physicochemical parameters are shown by red arrows, the Soil elements are illustrated by black arrows, the plant plant variables are illustrated by green arrows，the climatic factors are illustrated by blue arrows, differently colored shapes correspond to different samples from seven *Stipa* taxa. SOC: soil organic carbon; TN: total nitrogen; TK: total kalium; NO_3_^-^-N: nitrate nitrogen; AP: alkaline and neutral olsen-phosphorus; AK: available potassium; Fe: iron element; Cu: copper element; Zn: zinc element; Mn:manganese element; Ca: calcium element; S：sulfur element; VS：Shannon-Wiener index of plot; VC: plant coverage; SB: biomass of *Stipa*; CP: crude protein; EE: crude fat; CF: crude fibre; MAT: mean annual temperature; MAP: mean annual precipitation.


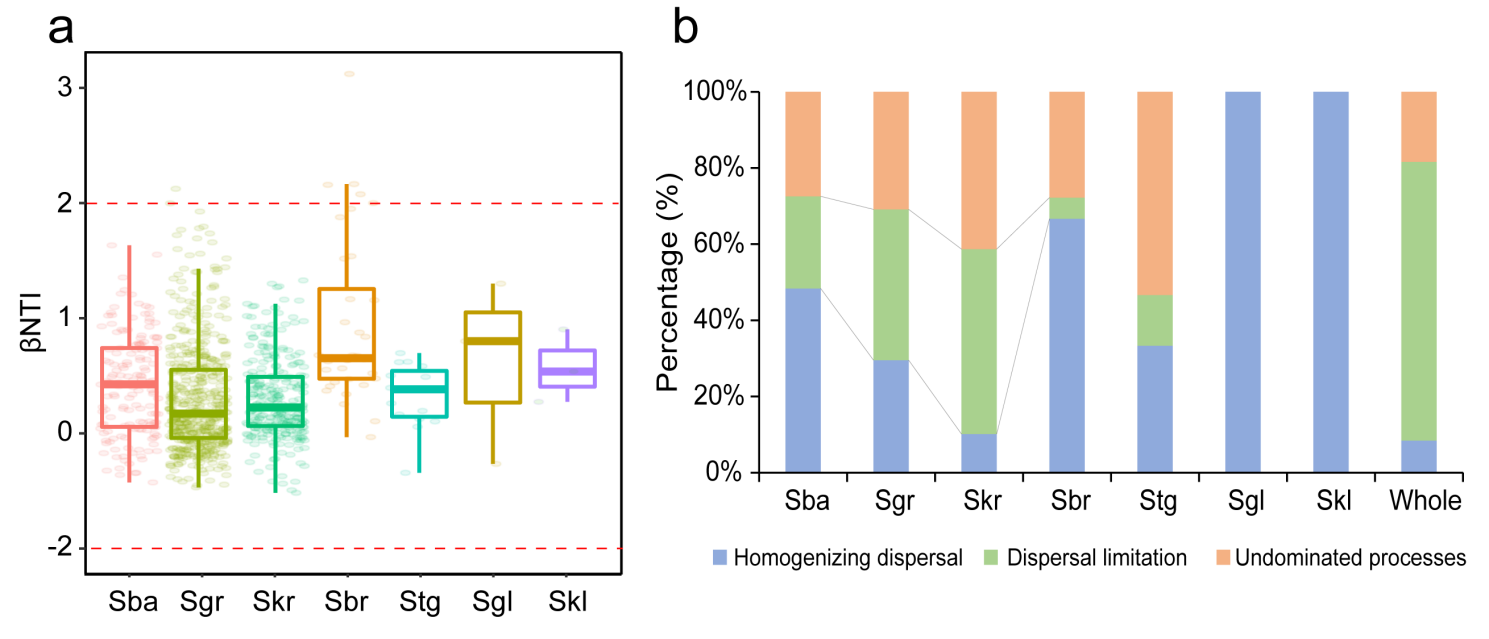


**Figure S3** | The bacterial community assembly processes across *Stipa* taxa root-zone soil. **a)** The values of the weighted beta nearest taxon index (βNTI) for root-zone soil bacterial communities present. Horizontal dashed red lines indicate upper and lower significance thresholds at βNTI = +2 and -2, respectively. The βNTI values > +2 (variable selection) or < −2 (homogeneous selection) means significantly more or less phylogenetic turnover than expected, respectively, indicating the predominance of deterministic processes. IF the |βNTI|≤ 2, this indicates that stochastic processes predominate. **b)** Relatively explained degree of community stochastic processes based on the values of RCBray; |βNTI| < 2 and RCBray < −0.95, |βNTI| < 2 and RCBray > 0.95, |βNTI| < 2 and |RCBray| <0.95 respectively indicate homogenizing dispersal, dispersal limitation and undominated processes (weak selection, weak dispersal, diversification, and/or drift ). Notes: *S. baicalensis* (Sba), *S. grandis* (Sgr), *S. krylovii* (Skr), *S. glareosa* (Sgl), *S. klemenzii* (Skl), *S. breviflora* (Sbr), and *S. gobica* (Stg).
